# A Scoping Review of Interventions to Address Financial Toxicity in Pediatric and Adult Patients and Survivors of Cancer

**DOI:** 10.1002/cam4.70879

**Published:** 2025-04-18

**Authors:** Christina Ping, D. Carolina Andrade, Ashley Housten, Michelle Doering, Eliana Goldstein, Mary C. Politi

**Affiliations:** ^1^ Washington University School of Medicine Saint Louis Missouri USA; ^2^ Department of Surgery, Division of Public Health Sciences Washington University School of Medicine Saint Louis Missouri USA; ^3^ Bernard Becker Medical Library Washington University School of Medicine Saint Louis Missouri USA; ^4^ School of Public Health Washington University in St. Louis Saint Louis Missouri USA

**Keywords:** financial stress, health expenditure, neoplasm, quality of life

## Abstract

**Background:**

Financial toxicity (FT) is a common and significant challenge for people with cancer, impacting immediate clinical outcomes such as treatment adherence, as well as long‐term outcomes such as quality of life and mortality. Multiple studies have tested interventions to address FT and develop recommendations for their implementation.

**Methods:**

In this scoping review, we analyzed thirty‐six studies across 35,405 participants examining institution‐based interventions for FT in both pediatric and adult patients and survivors of cancer in the U.S.

**Results:**

Common interventions included: financial navigation (*n* = 15), direct financial/medical assistance (*n* = 8), financial counseling or coaching (*n* = 5), and cost conversations prompters or encounter decision aids for treatment and cost (*n* = 5). Outcome measures varied widely, including the COmprehensive Score for financial Toxicity (COST), the Medical Expenditure Panel Survey (MEPS), total out‐of‐pocket costs or savings, and mental/psychological quality‐of‐life measured by the Patient‐Reported Outcomes Measurement Information System (PROMIS). Many interventions showed promising results on improving FT, including financial assistance (e.g., free medication, copay assistance), treatment and insurance decision aids, and financial counseling. These strategies improved FT‐related metrics, including patient out‐of‐pocket costs, care‐related financial burden, health insurance knowledge, quality of life, and even overall survival. There was no dominant intervention method, with both low‐ and high‐resource options proving effective.

**Discussion:**

Future research should seek to understand causal relationships between interventions and FT through robust study designs, such as randomized controlled trials with longitudinal follow‐up, and evaluate interventions' implementation potential. There is also a need for standardized metrics for evaluating and reporting FT to better compare different interventions' success.

## 1 Background

Financial toxicity (FT) is a common challenge for people with cancer. FT comprises the cost burden and associated stress from diagnosis, treatment, and follow‐up care. It includes both direct costs (e.g., copayments, coinsurance, and other out‐of‐pocket spending for procedures or medications) and indirect costs (e.g., travel, changes to employment, time, caregiving) of care. An estimated 80% of people with cancer will leave the workforce during initial treatment [1]; more than 40% of patients will deplete their savings in the first 2 years of treatment [1]; and 30% of Americans with a history of cancer report difficulty paying medical bills [2].

FT significantly impacts individuals' health and wellbeing, and individuals may experience FT at different levels of severity during treatment and survivorship. In the short term, individuals with high FT report challenges following recommended treatment plans, delaying, rationing, or avoiding needed care, and cutting back on other expenses to cover the cost of care [3, 4]. In the long term, those reporting high FT have an increased risk of mortality, decreased quality of life, increased risk of depression, and greater worry about cancer and its recurrence [5].

Multiple studies have developed and tested interventions to address FT and identify recommendations for their implementation. These interventions range in terms of accessibility, eligibility, design, level of engagement required from the patient, and clinical care setting. Comparing results across studies and populations in systematic or scoping reviews has been challenged by variable study designs, inclusion criteria, and outcomes. For example, in a review of five financial assistance programs to address FT among patients with cancer, only one study measured a reduction of patients' out‐of‐pocket costs, with the other four small non‐randomized studies focusing on feasibility and preliminary testing [6]. In a review of six financial navigation programs, analyses of efficacy were limited by small sample sizes and high attrition rates [7]. Similarly, in a recently published review of interventions for adult patients actively receiving treatment for cancer, focused inclusion criteria resulted in limited representation of some key populations, such as non‐White or male patients [8], inhibiting the review's ability to make broader inferences about the effectiveness of the FT interventions. These previously conducted reviews were also narrow in scope, examining either particular types of interventions (e.g., financial navigation), limiting their analysis of format, structure, duration, or long‐term impact on direct and indirect outcomes, or excluding important patient populations (e.g., pediatrics).

To build on others' foundational work, in this paper, we aim to further our understanding of FT interventions' effectiveness and implementation potential by conducting a scoping review of existing institutionally‐based FT interventions for patients with cancer. We characterized and compared study quality, impact, effectiveness, and implementation potential, using broad inclusion criteria for age and intervention type.

## 2 Methods

### 2.1 Search Strategy

**FIGURE 1:**
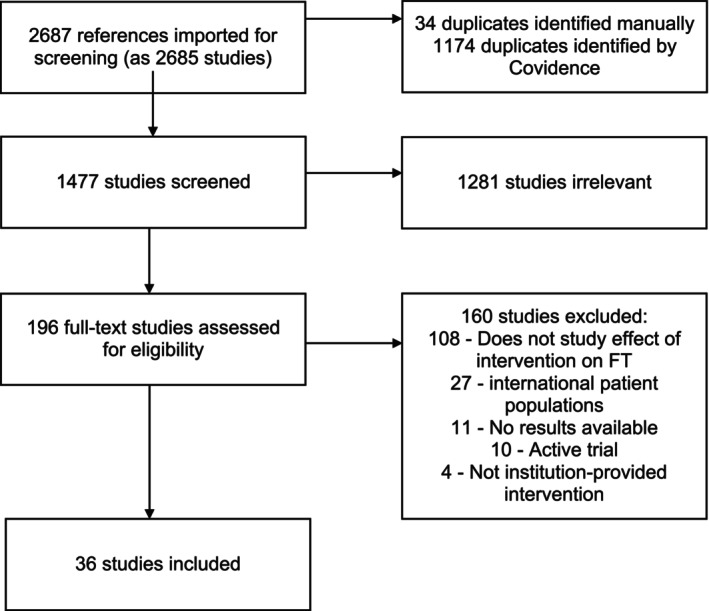
PRISMA diagram of articles screened and included in this review.

We searched the published literature for interventions to address FT in oncology using strategies created with a medical librarian. Search strategies were established using a combination of standardized terms and keywords and executed in the databases Ovid Medline, Embase.com, PubMed, Web of Science, and Clinicaltrials.gov in May 2024 using an English language filter and a date limit of January 2013–May 2024. Conference abstracts were excluded from the search. A total of 2687 citations were imported into Covidence, a screening and data extraction software for conducting reviews. Duplicate articles (*n* = 1174) were removed, for a total of 1513 unique citations (Figure 1). Full electronic search strategies are provided in the Data S1. An example of the search terms is included in the Data S1.

### 2.2 Article Selection Process

Two reviewers (CP, CA) independently screened all titles and abstracts for eligibility and relevance and resolved discrepancies between reviewers together, consulting with other authors (MP, AH) when needed. The fullext of screened articles was then evaluated on the following criteria: (1) if the full text was available in English, (2) if the study examined an intervention used to address FT in people with cancer or survivors, and (3) if the study explored factors that influence the success of different FT interventions. Figure 1 shows the article selection process. Cohen's kappa between CP and CA was 0.902 at the full‐text screening stage.

### 2.3 Data Extraction

Data extraction for included articles was completed by both reviewers (CP, CA) based on an approved extraction template created and reviewed by the study team (MP, AH). Consensus for extraction was obtained between both parties; if consensus was not reached, the study team was consulted.

## 3 Results

Table 1 details the characteristics of studies included in this review. Though interventions were tested among people with a variety of cancer types and ages, most targeted adult patients (*n* = 34/36) rather than pediatric patients or families (*n* = 4/36). Those that included pediatric patients did so in a larger study that encompassed adult patients as well. Two studies did not specify a patient participant age range [9, 10]. Interventions were primarily conducted at National Cancer Institute (NCI) Comprehensive Cancer Centers or academic centers (*n* = 23/36), though community hospitals and outpatient clinics were also included (*n* = 4/36). Interventions that employed digital technologies (e.g., mobile apps, video‐based intervention delivery) were noted to be more prevalent in recent years. Only 12 out of 36 included studies reported any information on household income of included participants. Most reported sociodemographic characteristics, but many were limited in the racial diversity of their participants.

**TABLE 1:** Description of study characteristics (*N* = 36 studies; *N* = 35,405 total participants across studies).

Year	Lead author	Title	Region of US where participants were recruited	Setting type where patients were recruited	Study design	Intervention type	Cancer type	Total number of participants (No.)	Participants in control group (No.)	Participants in intervention group (No.)	Patient population age range	Total duration of follow‐up	Frequency of intervention touch points	Total number of intervention touch points	Primary outcome for FT	Secondary outcomes
2015	Zafar	The utility of cost discussions between patients with cancer and oncologists	South	NCI Cancer Center	Cross sectional study	Cost conversations	Breast; lung; colorectal; prostate; pancreatic; other	300	—	300	Adult (18–65 years old); elderly (> 65 years old)	3 months	3 months	2	Impact of cost conversation on patient costs	Objective financial burden, financial distress, quality of life
2018	Shankaran	Pilot feasibility study of an oncology financial navigation program	Pacific Northwest	NCI Cancer Center	Single‐arm pilot feasibility study	Financial navigation	Not specified	34	—	34	Adult (18–65 years old); elderly (> 65 years old)	6 months	Monthly (or more frequently if needed)	10	No primary outcome related to FT; feasibility is the primary outcome of the study	Anxiety around cost of treatment, self‐reported financial burden
2018	Yezefski	Impact of trained oncology financial navigators on patient out‐of‐pocket spending	National	Community Hospital	Prospective cohort study	Financial navigation and assistance	Not specified	3572	—	3572	Not specified	Not specified	Not specified	Not specified	Direct patient OOP savings for care	Patient benefit support (e.g., savings from insurance enrollment, marketplace maximization, community assistance), overall impact on hospital revenue
2019	Seetasith	The impact of copay assistance on patient out‐of‐pocket costs and treatment rates with ALK inhibitors	National	IQVIA Formulary Impact Analyzer	Retrospective cohort study	Copay assistance	Lung	3143	2573	570	Adult (18–65 years old); elderly (> 65 years old)	1 year	N/A	N/A	Effect on OOP costs	Time to receipt of the drug, risk of prescription abandonment, risk of treatment discontinuation, persistence on treatment
2019	Lambert	Technology unlocks untapped potential in a financial navigation program	Midwest	Community Hospital	Observational study	Financial navigation	Breast; lung; colorectal; gastric; hematologic; gynecologic; bladder; head and neck	4616 screened, 244 identified as high priority	—	4616 screened, 244 identified as high priority	Adult (18–65 years old); elderly (> 65 years old)	8 months	Not specified	Not specified	Total approved savings by the financial navigation process	Community benefit (aid which benefits the patient directly and not necessarily the hospital), and revenue increase (aid that benefits the financial performance of the hospital)
2019	Sadigh	Pilot feasibility study of an oncology financial navigation program in brain cancer patients	South	NCI Cancer Center	Single‐arm feasibility study	Financial navigation	Breast; lung; colorectal; brain	12	—	12	Adult (18–65 years old)	9 months	Monthly	10 (baseline survey, monthly meetings for 6 months, follow‐up surveys every 3, 6, and 9 months)	Changes in mean financial toxicity score (COST)	Care non‐adherence, feasibility, type and amount of assistance provided to the participants
2019	Kircher	Piloting a financial counseling intervention for patients with cancer receiving chemotherapy	Midwest	NCI Cancer Center	Randomized prospective feasibility trial	Financial counseling	Lung; colorectal; gastric; soft tissue (e.g., sarcomas)	95	52	43	Adult (18–65 years old); elderly (> 65 years old)	2–5 months	Not specified	2	Financial distress via COST score (Primary outcome overall was feasibility)	Health‐related quality of life (measured by EORTC QLQ‐30), health insurance literacy (Health Insurance Literacy Measure), and acceptability of the intervention
2019	Siegel	Drug recovery and copay assistance program in a community cancer center: charity and challenges	South	Community Hospital	Observational study	Financial assistance	Not specified	Fiscal Year 2017–173 patients Fiscal Year 2018–256 patients	—	Fiscal Year 2017–102 copay assistance patients, 71 drug recovery patients Fiscal Year 2018–180 copay assistance patients, 76 drug recovery patients	Not specified	Not specified	Not specified	Not specified	Amount of copays and deductibles obtained from outside sources	Total acquisition cost of drugs recovered, patient drug recovery encounters (monthly drug recovery events per drug per patient)
2020	Politi	Encounter decision aids can prompt breast cancer surgery cost discussions: analysis of recorded consultations	Northeast; Midwest	NCI Cancer Center; Academic Center	Randomized controlled trial	Encounter decision aids	Breast	311	168	143	Adult (18–65 years old); elderly (> 65 years old)	No follow‐up	N/A	1	Frequency of cost conversations	Content of cost discussions, cost conversation initiator, length of cost conversation, referrals made to address cost
2020	Semin	Understanding breast cancer survivors' financial burden and distress after financial assistance	Midwest	Community organization	Cross sectional study	Financial assistance and resource navigation	Breast	118	—	118	Adult (18–65 years old); elderly (> 65 years old)	Not specified	Not specified	Not specified (avg. number of assistance touchpoints for the organization is 1.71)	Financial status, burden, and distress before, during, and after breast cancer treatment	Quality of life, impact of financial assistance on decision‐making, perceived social support
2020	Politi	Improving cancer patients' insurance choices (I Can PIC): a randomized trial of a personalized health insurance decision aid	Midwest	Academic Center; Community Hospital; Outpatient Clinics; Online advertisements, health and social service events	Randomized controlled trial	Health insurance decision aid	Breast; gastric; hematologic; gynecologic; prostate; bladder; skin; head and neck	263 enrolled and randomized, 206 completed the study	100	106	Adult (18–65 years old)	3–6 months	Not specified	2 (baseline and follow up)	Health insurance knowledge	Decision self‐efficacy, decisional conflict, financial toxicity, delay or avoidance of care
2020	Watabayashi	A pilot study of a comprehensive financial navigation program in patients with cancer and caregivers	Pacific Northwest	Academic Center	Prospective cohort study	Financial navigation	Not specified	30 patients, 18 caregivers	—	30 patients, 18 caregivers	Adult (18–65 years old); elderly (> 65 years old)	6 months	Monthly	9	General financial hardship (COST‐FACIT)	Caregiver burden (Caregiver Strain Index CIS), cost‐related nonadherence
2021	Fudzie	Impact of embedded medication assistance program specialists on medication access in outpatient oncology clinics	South	NCI Cancer Center	Retrospective cohort study	Medication assistance	Breast; lung; colorectal; gastric; hematologic; brain; soft tissue (e.g., sarcomas); gynecologic; prostate	305	—	305	Adult (18–65 years old); elderly (> 65 years old)	Not specified	Not specified	Not specified	Median prior authorization turnaround time	Total patient cost savings, median turnaround time for financial assistance programs
2021	Tarnasky	Mobile application to identify cancer treatment‐related financial assistance: results of a randomized controlled trial	South	NCI Cancer Center	Randomized controlled trial	Financial navigation	Breast; lung; colorectal; gastric; hematologic; gynecologic; prostate; bladder; kidney; melanoma; head and neck	200	100	100	Adult (18–65 years old); elderly (> 65 years old)	6 months	Every 3 months	4	OOP costs at 3 months	Financial distress measured using FACT‐COST, patient knowledge of financial resources specific to their care
2021	Farrugia	Financial counseling is associated with reduced financial difficulty scores in head and neck cancer patients treated with radiation therapy	Northeast	NCI Cancer Center	Observational study	Financial counseling	Head and neck	387	285	102	Adult (18–65 years old); elderly (> 65 years old)	Not specified	Not specified	Not specified	Financial difficulty score (EORTC QLQ‐C30)	Demographic variables were all assessed for a potential correlation with FT
2021	Raghavan	Levine cancer institute financial toxicity tumor board: a potential solution to an emerging problem	South	Academic Center	Observational Study	Financial toxicity tumor board	Not specified	Not specified	Not specified	Not specified	Not specified	Not specified	Not specified	Not specified	Not specified	Rate of FTTB resolution (%)
2022	Handley	A pilot feasibility study of digital health coaching for men with prostate cancer	Northeast	NCI Cancer Center	Single‐arm pilot feasibility trial	Digital health coaching	Prostate	88	—	88	Adult (18–65 years old); elderly (> 65 years old)	3 months	Weekly phone calls, up to 4 nudges per week	12 Phone calls, up to 48 nudges	N/A—primary outcome was feasibility, not FT‐related	COST score, self‐efficacy (measured using the Cancer Behavior Inventory), health‐related quality of life (using PROMIS Global Health), and prostate cancer‐specific quality of life (using EPIC‐CP)
2022	Charles	A case study of adapting a health insurance decision intervention from trial into routine cancer care	Midwest	NCI Cancer Center	Case series	Financial navigation	Lung; colorectal; gynecologic	136	68	68	Adult (18–65 years old); elderly (> 65 years old)	3–6 months	3 months	2	Health insurance knowledge	Health insurance literacy, frequency and type of cost conversation, financial toxicity, and patient referrals to resources
2022	Seymour	How to effectively decrease patient co‐payments of high‐cost drugs through innovation: lessons from the karmanos specialty pharmacy	Midwest	NCI Cancer Center	Retrospective cohort study	Financial assistance	Not specified	463	—	463	Not specified	1 year	N/A	N/A	Reduction in patient co‐pay	Time from prescription written to delivery, amount and type of FA obtained, and cost distribution between patients and payers
2022	Knight	Financial toxicity intervention improves outcomes in patients with hematologic malignancy	South	NCI Cancer Center	Single‐arm interventional trial	Screening, financial counseling, medication assistance	Hematologic	105	46	59	Adult (18–65 years old); elderly (> 65 years old)	1 year	Every 2 months	Minimum 3, maximum not specified	Changes in PROMIS score	Overall survival
2022	Sadigh	Treatment out‐of‐pocket cost communication and remote financial navigation in patients with cancer: a feasibility study	South	Outpatient Clinics	Single‐arm feasibility study	Financial navigation, cost conversations	Breast; lung; colorectal; gastric; prostate; bladder; kidney; testicular; other	23	—	23	Adult (18–65 years old); elderly (> 65 years old)	3 months	1–3 months	4^a^	Primary outcome of study was for feasibility, not FT	Enrollment in financial support services, approved monetary amount of assistance, financial worry, cost‐related care nonadherence, material financial hardship
2022	Hamel	The DISCO App: A pilot test of a multi‐level intervention to reduce the financial burden of cancer through improved cost communication	Midwest	Outpatient Clinics	Single‐arm feasibility study	Cost conversation prompting	Breast; lung; colorectal; prostate	32 patients, 3 physicians		32 patients, 3 physicians	Adult (18–65 years old); elderly (> 65 years old)	Assessments were immediately before and after clinic visit, no additional follow up	N/A	2	Cost discussions (frequency, initiator, and topics)	Patient self‐reported self‐efficacy for managing treatment costs, interacting with physicians, and treatment cost‐related distress
2023	Sadigh	Improving palbociclib adherence among women with metastatic breast cancer using a CONnected CUstomized Treatment Platform: A pilot study	West Coast	NCI Cancer Center	Single‐arm feasibility pilot	Financial counseling and pill‐tracker	Breast	29	—	29	Adult (18–65 years old); elderly (> 65 years old)	3 months	3 months	2	Primary outcome of study not related to FT given was feasibility trial	Financial worry (using COST score), QoL (using PROMIS‐10), adherence to palbociclib (using the PROMIS PMAS)
2023	Kirchhoff	Health insurance literacy improvements among recently diagnosed adolescents and young adults with cancer	Southwest	NCI Cancer Center; Academic Center	Randomized feasibility trial	Health insurance literacy navigation	Not specified	86	41	45	Adult (18–65 years old)	5 months	Every other week	4	Feasibility and acceptability, assessed using the number of completed sessions per participant	Health insurance literacy measured via four different measures, financial hardship (COST), perceived stress scale
2023	Ragavan	Impact of a comprehensive financial resource on financial toxicity in a national, multiethnic sample of adult, adolescent/young adult, and pediatric patients with cancer	National	Independent Foundation	Cross sectional study	Financial assistance	Breast; hematologic; brain; soft tissue (e.g., sarcomas); not specified; lymphoma, neuroblastoma, retinoblastoma	330	—	330	Pediatric (< 18 years old); adult (18–65 years old)	Not specified	Not specified	Not specified	Patient‐reported financial toxicity, through three items: (1) feeling of financial stress during the prior week, (2) feeling in financial control during the prior week, (3) feeling that expenses were met during the prior week	Confidence in Family Reach's intervention to decrease feelings of financial distress
2023	Edward	Coverage and cost‐of‐care links: addressing financial toxicity among patients with hematologic cancer and their caregivers	South	NCI Cancer Center	Single‐arm feasibility trial	Financial navigation	Hematologic	60 patients, 34 caregivers		60 patients, 34 caregivers	Adult (18–65 years old); elderly (> 65 years old)	Not specified	Not specified	Average of three in‐person meetings (range 0–21) and five telephone interactions (range 1–23)	Patient‐reported FT in three domains: psychological response, material conditions, and coping behaviors, measured using the COST survey and the MEPS‐ECSS (Medical Expenditure Panel Survey—Experiences with cancer Survivorship Supplement)	Distress, measured using the NCCN's DT; health‐related QoL using the PROMIS scales; feasibility, acceptability, and appropriateness
2023	Bello	The impact of social determinants of health, namely financial assistance, on overall survival in advanced‐stage non‐small cell lung cancer patients	South	Academic Center	Retrospective cohort study	Financial assistance	Lung	125	61	64	Adult (18–65 years old); elderly (> 65 years old)	7 years	N/A	N/A	Overall survival	N/A
2023	Thom	Financial toxicity order set: implementing a simple intervention to better connect patients with resources	Northeast	NCI Cancer Center	Quality improvement	EMR feature to refer patients directly for financial assistance	Not specified	22,578 out of 89,283 patients identified to be with FT, 670 unique patients received order		670	Pediatric (< 18 years old); adult (18–65 years old); elderly (> 65 years old)	12 months	N/A	Not specified	Financial assistance order indications and frequency	Amount of aid distributed for copayment and essential needs
2023	Politi	The impact of adding cost information to a conversation aid to support shared decision making about low‐risk prostate cancer treatment: results of a stepped‐wedge cluster randomized trial	Midwest	NCI Cancer Center	Stepped wedge cluster randomized trial	Cost conversation prompt	Prostate	117	51	66	Adult (18–65 years old); elderly (> 65 years old)	3 months	N/A	2	Decisional conflict	Decisional regret, treatment choice received, and financial toxicity, frequency of cost conversations and referrals to address costs
2023	Blinder	Financial toxicity monitoring in a randomized controlled trial of patient‐reported outcomes during cancer treatment (Alliance AFT‐39)	National	Outpatient Clinics	Randomized controlled trial	Digital symptom monitoring	Breast; lung; colorectal; gastric; hematologic; brain; soft tissue (e.g., sarcomas); gynecologic; prostate; bladder; kidney; liver; pancreatic; testicular; melanoma; skin	1191	598	593	Adult (18–65 years old); elderly (> 65 years old)	1 year or until treatment ended	Weekly survey, but only had an FT question once a month and the EORTC‐FT questionnaire every three months	12 FACIT‐COST, 6 EORTC surveys	Change in financial difficulties using the QLQ‐C30	FT screening FACIT‐COST (added 2019)
2023	Alacevich	A point‐of‐care pilot randomized intervention to connect patients with cancer‐induced financial toxicity to telehealth financial counseling	South	NCI Cancer Center	Randomized controlled trial	Financial counseling	Not specified	121	40	40 individual counseling, 41 group counseling	Adult (18–65 years old); elderly (> 65 years old)	3 months	2 weeks apart	3	Financial toxicity measured using the COST score	Telehealth Usability Questionnaire
2023	Thom	Using real‐world data to explore the impact of one‐time financial grants among young adult cancer survivors	National	Independent Foundation	Retrospective cohort study	Financial assistance	Breast; colorectal; hematologic; brain; gynecologic; thyroid	300	—	300	Adult (18–65 years old)	6 months	6 months	2	Change in financial well‐being	Change in access to care, health status, and quality of life
2024	Parikh	Lay healthcare worker financial toxicity intervention: a pilot financial toxicity screening and referral program	West Coast	NCI Cancer Center	Quality improvement	Financial screening and counseling	Prostate; bladder; kidney	185	—	185	Adult (18–65 years old); elderly (> 65 years old)	3 months	Not specified	2	N/A—feasibility was primary endpoint, though this is not an FT‐related endpoint	Change in financial burden scores, post‐intervention patient‐reported satisfaction with the consultation, assessment of financial resources and services offered to patients
2024	Bell‐Brown	A proactive financial navigation intervention in patients with newly diagnosed gastric and gastroesophageal junction adenocarcinoma	Pacific Northwest	NCI Cancer Center	Randomized controlled trial	Financial navigation and assistance	Gastric; esophageal	19 patients, 11 caregivers	9 patients, 8 caregivers	10 patients, 4 caregivers	Adult (18–65 years old); elderly (> 65 years old)	6 months	Monthly	5	Incidence of financial hardship, defined as follows: accrual of debt, income decline of â 20%, or taking loans to pay for treatment	Quality of life (Functional Assessment of Cancer Therapy—General), subjective financial distress (COST score), qualitative assessment of access to and use of financial assistance, caregiver quality of life (City of Hope Quality of Life Family Version), caregiver burden (social well‐being subscale of the City of Hope Quality of Life Questionnaire)
2024	Park	Health insurance navigation tools intervention: a pilot trial within the childhood cancer survivor study	National	NCI Cancer Center	Randomized controlled trial	Health insurance navigation	Hematologic; brain; soft tissue (e.g., sarcomas); bone, neuroblastoma, Wilms tumor, Hodgkin lymphoma, NHL	91 consented, 82 completed baseline surveys	41	41	Adult (18–65 years old); elderly (> 65 years old)	5 months	Biweekly	6	Health insurance literacy	Familiarity with ACA provisions, health insurance satisfaction, and psychological and behavioral financial hardship
2024	Edward	Financial‐legal navigation reduces financial toxicity of pediatric and AYA cancers	South	NCI Cancer Center	Single‐arm feasibility trial	Financial navigation, cost conversations	Not specified	61 (15 adult patients, 46 caregivers)	—	45 financial navigation only. 6 legal navigation only, 10 both financial and legal navigation	Pediatric (< 18 years old); adult (18–65 years old)	Not specified	Not specified	Not specified	Mean total financial toxicity score measured from three subscores for psychological response (COST), material conditions (MEPS‐ECSS), and coping behaviors (MEPS‐ECSS)	Health‐related QoL measured using the Patient‐Reported Outcomes Measurement Information System (PROMIS) physical and mental health subscales, the PROMIS Anxiety short form, and the PROMIS Depression short form; PROMIS Global Health Parent‐Proxy was used to measure child QoL where applicable; feasibility; acceptability; appropriateness

Abbreviations: ACA, Affordable Care Act; AYA, adolescent and young adult; COST, COmprehensive Score for Financial Toxicity; EORTC QLQ‐C30, European Organization for Research and Treatment of Cancer (EORTC) Quality of Life Questionnaire Core 30; EPIC‐CP, Expanded Prostate Cancer Index Composite for Clinical Practice; FT, financial toxicity; MEPS‐ECSS, Medical Expenditure Panel Survey‐Experiences with Cancer Survivorship Supplement; NCCN‐DT, National Comprehensive Cancer Network Distress Thermometer; NCI, National Cancer Institute; OOP, out of pocket; PROMIS, Patient‐Reported Outcomes Measurement Information System; PROMIS‐PMAS, PROMIS Medication Adherence Scale.

^a^ Eight patients were selected to complete an additional interview 1–3 months after the initial consultation, making it 5 touch points for this group.

Common intervention activities trialed included: financial counseling or coaching (*n* = 5), cost conversations prompters or encounter decision aids (*n* = 5), direct financial (e.g., copay) or medical (e.g., drugs) assistance (*n* = 8), and financial navigation, e.g., charity programs, grants, or patient assistance programs; financial and health literacy education (*n* = 15). The remaining three studies used digital symptom trackers, physician‐directed referrals for financial assistance, or an interdisciplinary financial toxicity tumor board, respectively.

Ten of the 36 studies were feasibility studies with limited statistical power for analyzing outcomes directly related to FT. An additional two studies were unable to conduct final analyses due to high levels of attrition in their patient sample populations. Ten studies employed randomized designs, with eight specifically being randomized controlled trials.

No substantial differences in FT outcomes were observed between the various delivery modalities, such as mobile versus in‐person or synchronous versus asynchronous, nor the method of assistance, such as direct cash grants versus medication samples versus financial counseling (Table 2). Among the studies included, there was wide variation in outcome measures that were used to define “financial toxicity”, including the COmprehensive Score for financial Toxicity (COST) score, the Medical Expenditure Panel Survey (MEPS), both total out‐of‐pocket costs or savings in dollars, and mental and psychological quality of life as measured by the Patient‐Reported Outcomes Measurement Information System (PROMIS).

**TABLE 2:** Primary outcomes and key findings related to FT of included studies.

Year	Lead author	Total number of participants	Number or percentage of participants who completed the intervention	Primary outcome	Control, *n* (range), *p* (when relevant)	Intervention, *n* (range), *p* (when available)	Key results from paper	Study limitations as reported by the paper
2015	Zafar	300	300	Impact of cost conversations on OOP costs	N/A	57% patients reported lower OOP costs because of a cost discussion	Patients vary in their desire to discuss costs with providers Most patients who spoke with doctors about costs believed it helped reduce their costs Patients more willing to discuss costs at 3‐months follow‐up Higher subjective financial distress was associated with a greater likelihood to desire a cost discussion Nonwhite race was associated with a lower likelihood to desire a cost discussion. Most of the time, costs were reduced without altering the care that was delivered	Potential selection bias from consecutive, non‐random sampling of patients with cancer, most of whom were treated at a major referral center Lack of validated measures for quantifying patient's desire to discuss costs Short follow up period Reliance on patient self‐report for outcome measures
2018	Shankaran	34	20 completed at least one component	No FT‐related primary outcome for study	N/A	N/A	Patients overall satisfied with the financial literacy course, coaching, and case managers Impact on financial toxicity or burden was not observed	Single institution study that primarily serves a white and insured patient population, limiting generalizability Lack of standardization of the type and scope of assistance that CENTS and PAF could provide Cost savings were not quantified Could not provide patients or coaches with precise out‐of‐pocket estimates for specific tests or treatments
2018	Yezefski	3572	3572	Mean OOP savings, per patient, across all 11 total follow‐up years for the 4 hospital sites, by source of savings	N/A	Free meds: $9,879,779 Premium assistance: $14,117,157 ($9,411,438—$18,822,876) Co‐pay assistance: $2,541,105	Financial navigators can help mitigate losses incurred by hospitals that treat large underserved populations by increasing the number of insured patients, paying insurance premiums to help keep patients insured, finding programs to help patients with OOP costs that would not otherwise be paid, and obtaining free medications from patient assistance programs Hospitals were able to avoid write‐offs and save on charity care by an average of $2.1 M per year.	Only hospitals that already used trained financial navigators were used Lack of data on utilization of financial navigation at the hospital sites prior to instituting the structured program No comparison to similar hospitals that did not use trained financial navigators Only “new” patients were included in the data set each year, minimizing the long‐term benefits of financial assistance Limited data on out‐of‐pocket costs for uninsured patients
2019	Seetasith	3143	N/A	Average final OOP cost	$1205 ($3543)	$26 ($229) (*p* < 0.001)	Copay assistance significantly reduced OOP payments, reduced the time until patients received their prescription, lowered the risk of abandoning ALKi treatment, and improved treatment persistence compared to those who did not receive copay assistance	Retrospective claims database, with potential for data entry errors Inability to confirm prescription pick‐ups outside the FIA network (which would impact estimates in differences in OOP costs, persistence, and adherence) Unobserved factors (such as patient financial circumstances) were not accounted for Patients that received copay assistance for a later claim (but not the first) were excluded, potentially lowering the generalizability of findings to other forms of financial assistance that require longer processing times
2019	Lambert	4616 screened, 244 identified as high priority	181	Total approved savings by the financial navigation process	N/A	$3,553,453 ($19,632 / patient)	Financial navigation software expanded the reach and delivery of program services and reduced administrative burden on program staff Navigators secured a combined total of $3,553,453 in savings; $1,524,562 of this savings accounted for community benefit; and $259,593 contributed to revenue increase (a direct benefit to the cancer center)	Study did not take place during an open enrollment period, which may have decreased the number of patients benefiting from insurance optimization
2019	Sadigh	12	2	Mean change in COST score at 3‐month follow‐up	N/A	Baseline: 8.8 3‐month follow‐up: 8.0 (*p* = 0.89)	Intervention did not show improvement in financial toxicity scores due to small sample size and high attrition rate	Difficulty recruiting and sustaining follow‐up with patients due to death, cognitive decline, and increasing caregiver dependency Contact initiation and consistency with PAF was challenging
2019	Kircher	95	13 out of 43 intervention group participants completed whole intervention and planned assessments	Mean COST score	25.5 (11.8)^a^	N/A	Feasible and acceptable for patients to be contacted by phone for a financial counseling (FC) discussion Completion of the two‐part intervention (phone and in‐person) was not feasible due to both patient and counselor barriers to completion of the in‐person visit Only 30% of patients randomly assigned to the FC arm received the full intervention (including both the phone call and the in‐person visit) OOP estimation tool was considered understandable and acceptable to the majority of participants No significant changes in financial distress were observed between the FC and standard care (SC) arms.	Conducted in a single institution in a large metropolitan area, limiting generalizability Low adherence diluted effect of intervention Lack of quantification for out‐of‐pocket costs for nonchemotherapy services Analysis limited to only one cycle of chemotherapy, thereby underestimating patient total OOP costs
2019	Siegel	FY 2017–102 copay assistance, 71 drug recovery FY 2018–180 copay assistance, 76 drug recovery Total—182 copay assistance, 147 drug recovery	N/A	Amount of copays and deductibles obtained from outside sources	N/A	FY2017—$189,523 FY2018—$244,804	Intervention resulted in nearly $3.5 million worth of drugs recovered annually and significant cost avoidance for the institution More patients received help under the internal intervention compared to the previous year with an external vendor	None provided by study team
2020	Politi	311	311	Proportion of consultations with cost conversations	44 out of 168	88 out of 143 (*p* < 0.001)	Decision aid containing a cost prompt increased the frequency of cost discussions, with surgeons initiating cost discussions more often than patients Decision aid triggered more cost conversations about early‐stage breast surgery; during these conversations, non‐surgery costs were also discussed (genetic testing, radiation, etc.) Statistically significant relationship between the presence of a cost discussion and insurance status, but no relationship between cost discussions and surgery choice	Lack of a priori power calculation for secondary data analysis and descriptive study Lack of documentation on whether patients followed through with referrals maid Potential results skewing as surgeons randomized to one study group had lower patient volumes than those in other groups due to unexpected events
2020	Semin	118	N/A—this is cross‐sectional, so only participants who received assistance were included	Mean score of financial situation (Likert scale, 1 = no financial issues to 5 = large amount of issues) before and after breast cancer diagnosis	N/A	Before: 2.68 (1.09) After: 3.74 (1.04) (*p* < 0.001)	Financial distress increased significantly during cancer treatment and remains high after treatment Financial assistance was perceived to improve quality of life and control over financial decisions, but does not lower financial distress back to precancer levels Lack of social support during cancer treatment was associated with higher post‐treatment distress.	Only included participants who received financial assistance Small sample size Low response rate Retrospective nature may lead to recall bias Results may not be generalizable to all breast cancer survivors or those outside the Midwestern USA
2020	Politi	263 enrolled and randomized, 206 completed the study	180	Mean health insurance knowledge score after 3–6 month f/u, measured using an 8‐item assessment, with higher scores indicating higher knowledge	74.86 (17.32)	82.07 (+18) (*p* = 0.002)	I Can PIC tool significantly improved health insurance knowledge, but there were no significant differences in decisional conflict, decision self‐efficacy, financial toxicity, or delays of care between the I Can PIC or control group	Small sample size from a limited number of recruitment sites Inability to pair study enrollment with variable open enrollment periods Short follow‐up period Relatively educated sample Did not engage clinicians or the health care system about care costs
2020	Watabayashi	30 patients, 18 caregivers	10 patients, 8 caregivers	Mean change in COST score at follow‐up for patients	N/A	Baseline: 20.95 Follow‐up: 18.04 (*p* = 0.63)	Feasible to enroll both patients and patient‐caregiver dyads cross a range of insurance types and income levels Financial hardship scores remained stable, despite the program providing significant financial assistance, especially to lower‐income participants (PAF secured $772 / household for those receiving assistance, and FR provided $647 / household).	Pilot study conducted at single institution that serves a largely White, urban, and insured patient population Lack of standardized protocols for CENTS, PAF, and FR interventions Potential recall bias for survey questions about the pre‐diagnosis period Low survey response rate
2021	Fudzie	305	N/A—retrospective study	Median turnaround time for prior authorization approval	N/A	24 h (90 h)	Embedding MAP specialists in oncology clinics resulted in efficient PA approvals, significant financial savings for patients, and timely access to medications	Potential inaccuracies in turnaround times due to manual EHR updates required (times may be longer than actual time) Absence of a baseline comparison for PA times prior to the MAP intervention
2021	Tarnasky	200	55 participants completed both time points' OOP cost survey, 64 participants completed the FACT‐COST Score	Primary outcome, change in OOP cost, data not available given high level of missing data	N/A	N/A	Significant missing data precluded a definitive assessment of the primary outcome Post hoc analysis suggested that intervention users were more likely to apply for and receive financial assistance compared to controls	High attrition rate with high rates of participants missing follow‐up data Possible bias due to retrospective post hoc analysis Limitations in generalizability due to specific inclusion criteria and study population
2021	Farrugia	387	102 out of 102 intervention participants	Change in percent of patients reporting each score for financial difficulty, 1 = not at all; 4 = very much, by score	One, not at all: 7.8% decrease Two: 1.4% increase Three: 4.9% increase Four, very much: 1.4% increase (*p* = 0.588)	One, not at all: 1% decrease Two: 3.9% increase Three: 2% decrease Four, very much: 1% decrease (*p* = 0.002)	Patients who did not undergo financial counseling (FC) had a significant increase in reported financial difficulty scores at the end of treatment No difference in pre‐ and post‐treatment scores in patients who had received FC FC is significantly associated with a − 0.2 units change in financial difficulty when adjusting for gender and nodal status Providing dedicated financial counseling resulted in stabilization of financial difficulty scores	Use of a single‐item question within the EORTC‐QLQ‐C30 to assess financial difficulty instead of comprehensive questionnaire Causes and consequences of financial toxicity are not characterized Potential selection bias in patients who received FC versus who did not Potential skewed results, as analysis did not account for median income, employment status, and education level among patients Lack of dedicated financial counselor employed by the institution
2021	Raghavan	Not specified	Not specified	Rate of FTTB Resolution (%), by case type	N/A	Uninsured or underinsured 43% Payer impediments 67% Coding or billing complexities 60% Precertification 100% Inadequate internal process 80%	Levine Cancer Institute's Financial Toxicity Tumor Board (FTTB) effectively reduced financial toxicity for people with cancer by addressing issues that typically increase their financial burden Board helped manage complex cases of financial toxicity through a multidisciplinary approach, involving various healthcare professionals and departments, and used proactive strategies such as a Patient Assistance Program for oncologic drugs to mitigate costs	Potential bias in the triage process that may skew towards more severe cases than what general oncology practices encounter
2022	Handley	88	63	N/A—primary outcome of study is not FT‐related	N/A	N/A	Digital health coaching for men with prostate cancer is feasible Most exploratory measures (self‐efficacy, COST score, quality of life measures) improved, but only changes in self‐efficacy and COST score were significant	Potential selection bias due to large proportion of email‐marketing‐recruited participants, and lack of racial and educational diversity among respondents Lack of acceptability surveys or exit interviews to formally evaluate acceptability Heterogeneity among treatment plans for patients limits ability to interpret PROs based on varying treatment's impacts on QoL Study was not designed to detect changes in patient‐reported outcomes
2022	Charles	136	N/A to study design	Mean health insurance knowledge	72.45	77.02	Patients consented to the study had relatively high baseline health literacy and low FT, both which were sustained throughout the study follow‐up period Intervention group saw a mild decrease in financial toxicity during the study period No statistically significant differences were seen between the interventions and control with regards to frequency of cost conversations, referrals to resources, or health literacy and knowledge	Non‐randomized study design Recruitment of historic controls Results may not be representative of lower‐income and/or racially diverse patients experiencing FT given the timing of recruitment (during COVID) and challenges of virtual recruitment during the pandemic
2022	Seymour	463	Not specified	Total reduction in patient co‐pay	N/A	$280,988 reduction (81% reduction in patient co‐pay amount)	Karmanos Specialty Pharmacy significantly reduced patient out‐of‐pocket expenses, primarily through foundation grants	Data on historical use of financial assistance, adherence, abandonment, patient‐reported financial hardship, household income, or socioeconomic factors were not collected Retrospective study design with population largely derived from a single academic cancer center Immunomodulatory drugs were not available through this specialty pharmacy and omitted from the cost for hematologic cancers Only looked at financial toxicity from drug
2022	Knight	105	59 out of 59 intervention group participants	Mean change in PROMIS scores by sub‐category	N/A	PROMIS Physical: 1.2 increase (*p* < 0.001) PROMIS Mental health: 1.0 increase (*p* < 0.001)	Screening for and intervening on FT is feasible in the outpatient hematologic cancer clinic setting, with improvements seen in patient quality of life (PROMIS) and overall survival measures	Single‐center, observational study without a planned control arm Potential recall bias from patient‐reported survey data Limited applicability of findings derived from a single center
2022	Sadigh	23	16	No FT‐related primary outcome	N/A	N/A	Remote delivery of personalized out‐of‐pocket cost communication, financial navigation, and counseling to patients with cancer in a community setting is feasible, with high completion rates and patient satisfaction Patient financial toxicity improved following the intervention Study also showed financial self‐efficacy was improved with intervention (not statistically, given study was not powered for this)	Small sample size Non‐randomized design Homogenous, non‐diverse population in the sample Short follow‐up period Lack of access to hospital‐negotiated prices for the price transparency program Only patients from a single clinic who could communicate in English were recruited Potential bias from the study coordinator and oncologists' experience with the intervention while facilitating recruitment
2022	Hamel	32 patients, 3 physicians	32 patients	Total number of cost conversations across all interactions	N/A	97 interactions	Multi‐level intervention is feasible and influences short‐term outcomes, including prompting treatment cost discussions and increasing patient self‐efficacy for interacting with physicians and managing costs DISCO App is innovative because it is the first to adapt the QPL, an effective paper‐based communication tool, into an electronic, individually tailorable, and scalable multi‐level intervention	Potential selection bias from single‐arm pilot with small sample size Patient refusal was not tracked, raising questions about generalizability and selection bias
2023	Sadigh	29	21	N/A—primary outcome was feasibility, not FT‐related	N/A	N/A	Mobile health intervention with a smartbox is feasible, with much higher adherence rates (95.8%) and sustained effect seen than reported in prior studies Financial worry significantly increased after three months, which is consistent with other studies in the metastatic setting which find worsening financial hardship over 12‐months	Small sample size Non‐randomized design Short follow‐period, limiting analyses of intervention effectiveness on adherence and other health outcomes Limited options in optimizing the smartbox design Study participants were recruited from a single clinic and required to communicate in English, limiting generalizability of findings
2023	Kirchhoff	86	37 out of 45 intervention group participants	Number of sessions completed by participants	N/A	No sessions—11 (25.5%) One session—3 (7%) Two sessions—2 (4.7%) Three sessions—0 All four sessions—29 (67.4%)	The intervention was feasible and acceptable, with significant improvements in HIL, insurance knowledge, ACA protections, and stress reduction in the intervention group compared to usual care Despite improvements in health literacy and perceived stress, there was no significant difference in financial toxicity observed between the two groups	Recruitment limited to hospitals in the Salt Lake City area Uninsured participants were not included Limited follow‐up period
2023	Ragavan	330	330	Primary outcome is regression model on factors predicting financial toxicity, not intervention's effect on FT	N/A	N/A	Financial assistance reduced financial stress for patients Hispanic, Black, and low‐income patients reported higher financial toxicity Receiving a higher amount of assistance was associated with increased confidence in reducing financial stress	Potential response bias, as survey was administered by Family Reach directly to participants Survey was not validated prior to implementation Lack of validated measure for measuring financial toxicity Survey was only administered in English Point‐in‐time estimate of financial toxicity, rather than longitudinal data
2023	Edward	60 patients, 34 caregivers	54 patients, 32 caregivers	Mean change in total FT score	N/A	Patients: 0.062 (0.29) (*p* = 0.13) Caregivers: 0.13 (0.34) (*p* = 0.041)	The only significant change from pre‐ to post‐intervention is in the psychological response score part of the COST score for patients Among caregivers, there was a significant improvement in COST across the subscales No significant differences between patients and caregivers on any of the measures except for PROMIS physical and mental health scores Patients and caregivers rated the intervention similarly on acceptability, appropriateness, and feasibility Financial navigator's services secured a total of $124,600 (~$2.5 k per participant).	Limited sample size Exclusion of non‐English speaking participants Lack of random assignment
2023	Bello	125	N/A—retrospective study	Median overall survival in years	0.545 (0.384–0.81)	1.01 (0.756–1.62) (*p* = 0.037)	Patients who received financial assistance were more likely to live longer (OR = 2.41, 95% CI 1.18, 4.96) Kaplan–Meier curves of survival show survival benefit extends out to 5 years	Missing data due to retrospective study design Recall bias for social determinant measures and comorbidities data Homogenous participant demographics, limiting study result generalizability
2023	Thom	22,578 out of 89,283 patients identified to be with FT, 670 unique patients received order	Not specified	Total number of orders placed	N/A	718 orders for 670 patients	The study established a need for a hospital‐level FT intervention in part 1, found that using the software was feasible and well‐received in part 2, and helpful to patients in part 3, resulting in $850 k of financial assistance	Study conducted at single urban comprehensive cancer center Lack of formal qualitative interviews of analyses of the experiencing of clinicians on the referral process FT referral orders were not associated with specific treatment plans or treatment modalities Limited pre‐ and post‐intervention comparisons Methodological changes between the two phases of the study
2023	Politi	117	46 of 51 control participants, 52 of 66 interventional participants	% of patients reporting decisional conflict, measured using SURE	Yes conflict—17.4% No conflict—82.6%	Yes conflict—9.8% No conflict—90.2% (*p* = 0.16)	The intervention did not significantly impact frequency of cost conversations, frequency of referrals for resources from surgeons, patient reported financial toxicity, or shared decision‐making Intervention increased deliberation among patients on treatment options, as evidenced by the reversal in percent undecided between the two time points	Small sample size with limited racial/ethnic diversity Heterogenous patient numbers by clinician, limiting statistical power to detect differences between them Recruitment challenges and disruptions due to the COVID‐19 pandemic
2023	Blinder	1191	295 out of 593 intervention participants	Percentage of patients reporting worsening financial difficulties	224 of 574 (39%)	173 of 572 (30.2%) (*p* = 0.004)	Overall, statistically significantly less participants at PRO practices developed worsened financial difficulties, compared with control practices Among patients who participated in the study after March 2019 (i.e., were screened for FT at least once if treated at a PRO practice), worsening of financial difficulties was significantly reduced for patients at PRO practices compared with control practices Based on the percentage of screened vs. unscreened patients developing FT, the number needed to screen is 11.4 Among Black patients, there was no difference in worsening of financial difficulties by arm.	Results for the primary study outcome (overall survival) were not available at time of report Potential confounding as FT screening was included as part of a larger intervention; possibility that routine remote symptom monitoring may have an impact on financial difficulties even without particular focus on FT Limited replicability of findings due to use of a single‐item measure as the primary outcome Lack of standardized approach to managing FT among sites Lack of data on the components of the patient‐clinician interactions that led to improvement in financial difficulties versus those that did not
2023	Alacevich	121	9 out of 81 intervention group participants, 20 out of 40 control group participants	Difference in mean COST score, absolute point change	2.5‐point increase (6.4) (*p* = 0.136)	Individual counseling—6.3‐point increase (11.6) (*p* = 0.5) Group counseling—5.8‐point increase (8.5) (*p* = 0.156) Individual + Group counseling – 6‐point increase (8.9) (*p* = 0.074)	Recruitment and randomization goals for the pilot study were feasible, while retention was much more difficult than anticipated Participants in both individual and group telehealth counseling sessions reported positive feedback, with slightly higher satisfaction reported by patients who received individual counseling (not statistically significant) Directional improvements in FT were observed, but nothing statistically significant	High attrition Small sample size and limited statistical power High study drop‐out rates
2023	Thom	300	300	Mean change in ability to pay expenses baseline vs. 6‐month follow‐up (Likert scale, 1 = strong agreement)	N/A	Baseline: 3.2 6‐month follow‐up: 2.8 (*p* < 0.001)	Modest amounts of one‐time financial support grants for YA cancer survivors can be impactful in improving overall financial well‐being and access to health care Grants are not cure‐alls for the patient conditions, as respondents still report credit card debt and psychological burden	Potential selection bias as a substantial amount of data was missing at the 6‐month follow‐up time, precluding analysis of the full sample's experience Findings reported only reflect the experience of grant recipients, leading to limited generalizability Lack of psychometrically tested measures of the outcomes of interest Exclusion of detailed data collection to insurance type and disability status Self‐selecting sample
2024	Parikh	185	18/18 received SW consultation	N/A—primary outcome was feasibility, not FT‐related	N/A	N/A	This multidisciplinary financial toxicity intervention using a lay health worker and social worker was feasible, acceptable, and associated with reduced financial burden among patients with advanced stages of urologic cancers	Limited generalizability from limited racial/ethnic diversity in sample and recruitment from a single‐institution academic center where most individuals reported annual household incomes of greater than $100,000 US dollars Small sample size Potential non‐response bias Unclear if difference detected in COST score has clinical significance since the full validated tool was not used Lack of analysis into whether intervention improved financial toxicity Potential response bias from follow‐up research assistance telephone interview
2024	Bell‐Brown	19 patients, 11 caregivers	8/12 usual group participants and 5/18 intervention group participants	Number of participants who reported events of developing financial hardship	3 out of 8	2 out of 5	Patients and caregivers were less likely to experience financial hardship at 6‐months post‐enrollment and less likely to experience a decrease in QoL at 3‐months post‐enrollment No statistically significant results were observed in patient‐specific outcomes related to FT or burden, nor any clinically meaningful decreases in caregiver QoL and financial burden Usual care patients were more likely to experience financial hardship and declines in quality of life compared to intervention patients Caregivers in both arms reported increased financial stress and poorer quality of life over the study period	Small number of participants High rate of participant decline Conducted at a single institution that primarily serves white, higher income, and insured population, limiting generalizability of the results
2024	Park	91 consented, 82 completed baseline surveys	33 out of 41 completed all 4 sessions	Mean change in health insurance literacy (lower scores are better), measured using 16‐items denoting confidence in understanding insurance terms and performing health insurance related activities	1.8‐point decrease (7.9)	9.1‐point decrease (7.6)	The intervention significantly improved health insurance literacy, knowledge of ACA provisions, satisfaction with health insurance, and reduced psychological financial hardship Behavioral financial hardship (such as not skipping medical care because of costs) did not improve at the 5‐month follow‐up	Short follow‐up time period Limited racial and ethnic minority representation Relatively well‐educated patient sample
2024	Edward	61 (15 adult patients, 46 caregivers)	61	Change in mean total FT score	N/A	Adult patients: 0.17‐point increase (0.34) (*p* = 0.12) Caregivers: 0.14‐point increase (0.26) (*p* = 0.001)	Participation in the program resulted in improvements in the psychological response domains of FT, as well as improved physical health for adult patients and parent‐proxy Global Health scores for pediatric patients	Small sample size Limited long‐term follow‐up Virtual points of contact with lawyers due to the pandemic (vs. in‐person for medical assistance)

Abbreviations: ACA, Affordable Care Act; ALKi, ALK inhibitors; CENTS, Consumer Education and Training Services; COST, Comprehensive Score for financial toxicity; EORTC‐QLQ‐C30, European Organization for Research and Treatment of Cancer Quality of Life Questionnaire Core 30; FIA, Formulary Impact Analyzer; FR, Family Reach; FT, financial toxicity; HIL, health insurance literacy; MAP, medication assistance program; OOP, out‐of‐pocket; PA, prior authorization; PAF, Patient Advocate Foundation; PROs, patient reported outcomes; QoL, quality of life.

^a^ No statistically significant change from baseline was found in the COST, InCharge, or EORTC single‐item measures between the interventional and control arms. The study paper reported values for the mean and standard deviation at baseline of all patients (control and intervention).

Despite the limited evidence of interventions on FT specifically, studies were able to observe statistically significant impacts on secondary and tertiary outcomes related to FT. Kirchhoff et al. [11] found that the virtual health insurance navigation platform (HIAYA‐CHAT) significantly improved health insurance literacy, health insurance terminology, and knowledge of the Affordable Care Act provisions in adolescents and young adults with patients. Semin et al. [12] and Knight et al. [13] both saw significant effects of financial screening, navigation, and resource support on patient quality of life. Seetasith et al. [14] observed that copay assistance for ALK‐inhibitors improved patient adherence, decreased treatment abandonment rates, and decreased patient out‐of‐pocket spending in people with non‐small cell lung cancer. And finally, Bello et al. [15] found an overall survival benefit in people with advanced stage non‐small‐cell lung cancer correlated with receiving financial assistance.

Some interventions proposed novel solutions to addressing FT, such as the development of a Financial Toxicity Tumor board [9], a smart pillbox and mobile reminder app duo paired with referral to financial navigation services when adherence issues were recorded [16], innovative “hosts” of FT interventions, such as the pharmacy [17, 18], combined medical and legal dual assistance approaches [19], and electronic medical record (EMR)‐based referral systems for FT support [20].

Table 3 ranks the intervention types trialed by impact on FT (high, medium, low) based on the currently available data detailed in this review. Examples of high impact interventions included cost or medication assistance, cost conversations, and multidisciplinary approaches to addressing FT (e.g., dual legal and financial assistance). Examples of medium‐to‐low impact interventions included financial navigation only or financial literacy/health insurance literacy education programs.

**TABLE 3:** Qualitative ranking of interventions by efficacy on financial toxicity.

Impact on FT‐related metrics (high, medium, low)^a^	Level of resource investment required (high, medium, low)^b^	Intervention type	Relevant studies
High	Low	Cost conversations	Zafar et al. (2015) [21] Politi, Yen et al. (2020) [20] Hamel et al. (2022) [22] Sadigh et al. (2022) [23] [16] Politi et al. (2023) [24]
High	Medium	Copay or medication assistance	Yezefski et al. (2018) [10] Seetasith et al. (2019) [14] Siegel et al. (2019) [25] Semin et al. (2020) [12] Fudzie et al. (2021) [17] Seymour et al. (2022) [18] Bello et al. (2023) [15] Ragavan et al. (2023) [26] Thom et al. (2023) [20]
High	High	Multidisciplinary navigation	Edward et al. (2024) [19]
Medium	High	Financial coaching or counseling	Kircher et al. (2019) [27] Farrugia et al. (2021) [28] Handley et al. (2022) [29] Knight et al. (2022) [13] Sadigh et al. (2023) [16] Alacevich et al. (2024) [30]
Medium	High	Financial toxicity tumor board	Raghavan et al. (2021) [9]
Medium	Medium	Ongoing financial toxicity monitoring with referral for patients with threshold scores	Blinder et al. (2023) [31]
Medium‐Low	Medium	Financial navigation only	Shankaran et al. (2018) [32] Lambert et al. (2019) [33] Sadigh et al. (2019) [34] Watabayashi et al. (2020) [35] Bell‐Brown et al. (2024) [36] Parikh et al. (2024) [37]
Medium‐Low	Medium‐Low	Financial literacy or health literacy education	Charles et al. (2022) [38] Politi, Grant et al. (2020) [39] Watabayashi et al. (2020) [35] Tarnasky et al. (2021) [40] Edward et al. (2023) [41] Kirchhoff et al. (2024) [11] Park et al. (2024) [42]

^a^ High, medium, and low designations were made based on the sample size of patients included in the study and the level of evidence for effectiveness on either direct (e.g., COST) or indirect (e.g., frequency of cost conversations) metrics related to FT.

^b^ High, medium, and low designations were made based on whether additional staff would be required for implementation, the level of involvement required for each patient, and the number of touchpoints with patients.

## 4 Discussion

We conducted a scoping review of 36 studies of 35,405 total participants and found that multiple interventions showed promise in improving FT among patients and survivors of cancer. These interventions include direct (e.g., copay assistance) and indirect (e.g., free medication) financial assistance [12, 13, 14, 15, 43], decision aids prompting cost discussions for treatment or insurance support [39, 44], financial counseling [28], health coaching on a variety of relevant topics, such as diet and lifestyle, treatment management, financial well‐being, and medication adherence [29], and digital symptom tracking [31]. These studies were able to demonstrate significant impacts on FT‐related metrics, such as patient out‐of‐pocket costs [14], financial burden and distress [12, 28, 31, 43], health insurance knowledge and confidence [39] or quality of life [13], and even direct clinical outcomes, such as overall survival [15]. Of note, all of the studies reporting positive improvements in FT‐related metrics included larger patient sample sizes (> 100 participants), and the trials for decision aids and digital symptom tracking were evaluated by randomized controlled study designs. These findings suggest that there are multiple effective methods to improve FT, offering institutions more options for implementation based on outcomes of interest and feasibility, as well as offering patients broader flexibility in choosing interventions that best suit their needs.

This review builds on previous work by broadening the patient population to include both pediatric and adult patients as well as survivors by examining the impact of FT on key patient‐centered outcomes. Furthermore, by expanding beyond financial navigation or assistance programs, this review was able to include interventions that took innovative approaches to addressing FT. It also ranks the interventions included in this review qualitatively by impact on FT‐related metrics and level of resources required to implement (Table 3). This review was able to identify simple and lower‐cost interventions that had comparable impact on FT‐related metrics to more resource‐intensive interventions. For example, digital and asynchronous delivery modalities could be more feasibly implemented without additional staff demands. Cost conversation prompts could also be implemented by existing clinical teams and integrated into their current practice without additional services or personnel. For lower‐resourced institutions, these interventions could be more feasible to implement to address FT among patients with cancer.

These findings should be considered in the context of the limitations of the current literature. First, a third of the included studies were either feasibility studies with limited ability to comment on the effectiveness of the interventions on improving FT or suffered from high rates of attrition impairing final analyses of gathered data. Second, there are currently no standardized guidelines for reporting FT nor benchmark thresholds for meaningful levels of change in FT. For example, the COST score is a 12‐item patient‐reported questionnaire that evaluates FT related to medical conditions, with statements scored on a Likert scale from 0 to 4 [45]. It is the most widely used measure of FT but is not routinely used in clinical care. Meanwhile, PROMIS assesses patient health status across physical, mental, and social domains and is more widely used in clinical settings [46]. Patient scores are compared versus scores of the general population as a reference, with varying cut‐off scores for normal limits depending on the specific metric being evaluated; however, PROMIS does not specifically evaluate FT. A third commonly used metric in the studies included in our paper was MEPS, which collects data on the cost and use of healthcare and health insurance from three sources (individual households, insurance carriers, and medical providers) [47]. MEPS provides more cost specificity and detail but does not as readily capture the burden of care costs on patients. The variations in target population (survivors vs. patient vs. parental or caregiver proxy) and metrics make comparisons between study results difficult as findings are not directly translatable. We recommend that future work identify the most robust and implementable measure(s) to evaluate FT and better compare interventions' effectiveness.

Third, most studies did not have diverse study populations in terms of race, cultural background, or primary language, and did not specifically recruit for patients most susceptible to FT. The majority of studies primarily included people who identified as White, educated, and English‐speaking; these individuals might be less susceptible to FT as they face fewer system‐level constraints [48]. Participant household income was reported only in 12 out of 36 studies in this review, which also limits evaluation of whether any observed effects on FT would hold true in the primary population that is expected to suffer from FT. Parikh et al. [37] even mentions that the majority of their patients reported household incomes > $100,000; these individuals might not be most affected by FT because they have greater financial resources. Patient age is also an important factor to consider when evaluating FT given that patients < 65 in age have been previously shown to be more affected by FT than those > 65 years [49, 50]. Furthermore, many longitudinal studies included in the review showed high rates of attrition. This was partly noted to be due to intrinsic factors of disease (e.g., a study including patients with metastatic brain cancer witnessed high mortality rates within the end‐stage population driving their attrition) as well as extrinsic factors (e.g., the COVID‐19 pandemic). Separately, however, patients who experience FT are also more likely to exhibit behaviors associated with care drop‐off, such as noncompliance or nonadherence to therapy [3, 4]. Lastly, it is important to recognize that while all of these interventions aim to improve FT, none actually address the root causes of systemic racism and disparities in poverty and access that underlie FT itself. Achieving this would require not just institutional interventions, but rather system‐level changes in healthcare cost, delivery, and social policies.

This review identified several areas for future work. One, there is a need for standardized metrics on evaluating FT and reporting out to better compare the success of different interventions. Two, there is a need to better understand statistical and causal relationships between institution‐based interventions and FT; randomized controlled designs with longitudinal follow‐up (at least 12–24 months) could better address these design limitations of some existing studies. Three, there is a need for research into addressing why there are high rates of attrition. Implementing retention strategies could support long‐term use and evaluation of FT interventions that are both feasible and effective. Longitudinal follow‐up and retention may be particularly difficult in populations which experience social limitations (such as those experiencing FT), so interventions need to consider ways to address these challenges, such as using multiple contact methods and ensuring intervention and repeated measurement strategies are accessible. These strategies are critical to improve both intervention effectiveness for the most impacted populations as well as the representativeness of data about interventions addressing FT for people with cancer. Finally, future work should consider subgroup‐specific dynamics (e.g., different types of cancers, age of the target patient population) to understand how the effectiveness of interventions may differ between these different subpopulations of patients with cancer and survivors, and ultimately allow us to design and tailor interventions toward the needs of particular groups.

## 5 Conclusion

In this scoping review, we examined thirty‐six interventions'studies designed to address FT. Though many studies were limited by their small sample sizes and attrition, promising results were seen both in interventions that provided immediate support, such as financial assistance or navigation, as well as those which focused on equipping patients with longer‐term skills, such as decision aids with cost prompts and virtual health literacy education. Asynchronous or digital delivery models and simple cost prompts were similarly effective compared to in‐person, clinic‐based interventions requiring additional personnel or novel programs; these interventions might help reduce FT with fewer resources required by institutions. Further randomized controlled studies with longer follow‐up periods and larger sample sizes are needed to better understand how interventions may affect FT and adjacent clinical outcomes among people with cancer both immediately and longitudinally. While institution‐based interventions can effectively impact patients' abilities to navigate the health system and receive supplemental support, ultimately, larger system‐level changes are needed to rectify underlying root causes contributing to FT.

## Author Contributions

**Christina Ping:** data curation (equal); formal analysis (lead); investigation (equal); visualization (lead); writing – original draft preparation, revision (lead); writing – review and editing (equal); **D. Carolina Andrade:** data curation (equal); formal analysis (supporting); investigation (equal); visualization (supporting); writing – review and editing (equal); **Ashley Housten:** conceptualization (equal); funding acquisition (equal); methodology (equal); supervision (equal); writing – review and editing (equal); **Michelle Doering:** resources (lead); **Eliana Goldstein:** writing – review and editing (equal); **Mary C. Politi:** conceptualization (equal); funding acquisition (equal); methodology (equal); supervision (equal); writing – review and editing (equal).

## Conflicts of Interest

Dr. Politi was a consultant for UCB Biopharma in 2024 and EpiQ Inc. in 2023 on topics unrelated to the content of this manuscript.

## Supporting information


Data S1.

## Data Availability

No new data were generated or analyzed in this review study; therefore, data sharing is not applicable.

## References

[1] A. M. Gilligan , D. S. Alberts , D. J. Roe , and G. H. Skrepnek , “Death or Debt? National Estimates of Financial Toxicity in Persons With Newly‐Diagnosed Cancer,” American Journal of Medicine 131, no. 10 (2018): 1187–1199.e5, 10.1016/j.amjmed.2018.05.020. 29906429

[2] M. P. Banegas , G. P. Guy , J. S. de Moor , et al., “For Working‐Age Cancer Survivors, Medical Debt and Bankruptcy Create Financial Hardships,” Health Affairs 35, no. 1 (2016): 54–61, 10.1377/hlthaff.2015.0830. 26733701 PMC6057727

[3] A. I. Neugut , M. Subar , E. T. Wilde , et al., “Association Between Prescription co‐Payment Amount and Compliance With Adjuvant Hormonal Therapy in Women With Early‐Stage Breast Cancer,” Journal of Clinical Oncology 29, no. 18 (2011): 2534–2542, 10.1200/JCO.2010.33.3179. 21606426 PMC3138633

[4] S. Kaul , J. C. Avila , H. B. Mehta , A. M. Rodriguez , Y. F. Kuo , and A. C. Kirchhoff , “Cost‐Related Medication Nonadherence Among Adolescent and Young Adult Cancer Survivors,” Cancer 123, no. 14 (2017): 2726–2734, 10.1002/cncr.30648. 28542734

[5] H. P. Kale and N. V. Carroll , “Self‐Reported Financial Burden of Cancer Care and Its Effect on Physical and Mental Health‐Related Quality of Life Among US Cancer Survivors,” Cancer 122, no. 8 (2016): 283–289, 10.1002/cncr.29808. 26991528

[6] S. Coughlin , L. Dean , and J. Cortes , “Financial Assistance Programs for Cancer Patients,” Current Cancer Reports 3, no. 1 (2021): 119–123, 10.25082/CCR.2021.01.007. 34568835 PMC8462924

[7] M. J. Doherty , B. Thom , and F. Gany , “Evidence of the Feasibility and Preliminary Efficacy of Oncology Financial Navigation: A Scoping Review,” Cancer Epidemiology, Biomarkers & Prevention 30, no. 10 (2021): 1778–1784, 10.1158/1055-9965.EPI-20-1853. PMC9022465 34341051

[8] S. Villalona , B. S. Castillo , C. Chavez Perez , et al., “Interventions to Mitigate Financial Toxicity in Adult Patients With Cancer in the United States: A Scoping Review,” Current Oncology 31, no. 2 (2024): 918–932, 10.3390/curroncol31020068. 38392062 PMC10888212

[9] D. Raghavan , N. A. Keith , H. R. Warden , et al., “Levine Cancer Institute Financial Toxicity Tumor Board: A Potential Solution to an Emerging Problem,” JCO Oncology Practice 17, no. 10 (2021): e1433–e1439, 10.1200/OP.21.00124. 34101495 PMC8791826

[10] T. Yezefski , J. Steelquist , K. Watabayashi , D. Sherman , and V. Shankaran , “Impact of Trained Oncology Financial Navigators on Patient Out‐Of‐Pocket Spending,” American Journal of Managed Care 24, no. 5 Suppl (2018): S74–S79. 29620814

[11] A. C. Kirchhoff , K. M. van Thiel Berghuijs , A. R. Waters , et al., “Health Insurance Literacy Improvements Among Recently Diagnosed Adolescents and Young Adults With Cancer: Results From a Pilot Randomized Controlled Trial,” JCO Oncology Practice 20, no. 1 (2024): 93–101, 10.1200/OP.23.00171. 38060990 PMC10827289

[12] J. N. Semin , D. Palm , L. M. Smith , and S. Ruttle , “Understanding Breast Cancer Survivors' Financial Burden and Distress After Financial Assistance,” Supportive Care in Cancer 28, no. 9 (2020): 4241–4248, 10.1007/s00520-019-05271-5. 31900619

[13] T. G. Knight , M. Aguiar , M. Robinson , et al., “Financial Toxicity Intervention Improves Outcomes in Patients With Hematologic Malignancy,” JCO Oncology Practice 18, no. 9 (2022): e1494–e1504, 10.1200/OP.22.00056. 35709421

[14] A. Seetasith , W. Wong , J. Tse , and C. Burudpakdee , “The Impact of Copay Assistance on Patient Out‐Of‐Pocket Costs and Treatment Rates With ALK Inhibitors,” Journal of Medical Economics 22, no. 5 (2019): 414–420, 10.1080/13696998.2019.1580200. 30729850

[15] A. Bello and N. S. Makani , “The Impact of Social Determinants of Health, Namely Financial Assistance, on Overall Survival in Advanced‐Stage Non‐Small Cell Lung Cancer Patients,” Cureus 15, no. 3 (2023): e36355, 10.7759/cureus.36355. 37082487 PMC10112388

[16] G. Sadigh , J. L. Meisel , K. Byers , et al., “Improving Palbociclib Adherence Among Women With Metastatic Breast Cancer Using a CONnected CUstomized Treatment Platform: A Pilot Study,” JCO Oncology Practice 29, no. 8 (2023): 1957–1964, 10.1177/10781552231161823. PMC10483024 36883245

[17] S. S. Fudzie , B. Luong , S. J. Jean , and S. J. Francart , “Impact of Embedded Medication Assistance Program Specialists on Medication Access in Outpatient Oncology Clinics,” Journal of Oncology Pharmacy Practice 27, no. 8 (2021): 1829–1834, 10.1177/1078155220970269. 33121352

[18] E. K. Seymour , L. Daniel , E. Pointer , J. Julian , S. T. Smith , and C. A. Schiffer , “How to Effectively Decrease Patient co‐Payments of High‐Cost Drugs Through Innovation: Lessons From the Karmanos Specialty Pharmacy,” JCO Oncology Practice 18, no. 1 (2022): e137–e151, 10.1200/OP.21.00207. 34406816

[19] J. Edward , K. D. Northrip , M. K. Rayens , et al., “Financial‐Legal Navigation Reduces Financial Toxicity of Pediatric and AYA Cancers,” JNCI Cancer Spectrum 8, no. 3 (2024): pkae025, 10.1093/jncics/pkae025. 38552323 PMC11087728

[20] B. Thom , S. Sokolowski , N. R. Abu‐Rustum , et al., “Financial Toxicity Order Set: Implementing a Simple Intervention to Better Connect Patients With Resources,” JCO Oncology Practice 19, no. 8 (2023): 662–668, 10.1200/OP.22.00669. 37319394 PMC10424913

[21] S. Y. Zafar , F. Chino , P. A. Ubel , et al., “The Utility of Cost Discussions Between Patients With Cancer and Oncologists,” American Journal of Managed Care 21, no. 9 (2015): 607–615. 26618364

[22] L. M. Hamel , D. W. Dougherty , T. A. Hastert , et al., “The DISCO App: A Pilot Test of a Multi‐Level Intervention to Reduce the Financial Burden of Cancer Through Improved Cost Communication,” PEC Innovation 1, no. 9918367980406676 (2022): 100002, 10.1016/j.pecinn.2021.100002. 37364004 PMC10194252

[23] G. Sadigh , D. Coleman , J. M. Switchenko , J. O. Hopkins , and R. C. Carlos , “Treatment Out‐Of‐Pocket Cost Communication and Remote Financial Navigation in Patients With Cancer: A Feasibility Study,” Supportive Care in Cancer 30, no. 10 (2022): 8173–8182, 10.1007/s00520-022-07270-5. 35796885 PMC9834906

[24] M. C. Politi , R. C. Forcino , K. Parrish , et al., “The Impact of Adding Cost Information to a Conversation Aid to Support Shared Decision Making About Low‐Risk Prostate Cancer Treatment: Results of a Stepped‐Wedge Cluster Randomised Trial,” Health Expectations 26, no. 5 (2023): 2023–2039, 10.1111/hex.13810. 37394739 PMC10485319

[25] R. D. Siegel , R. G. Slough , H. E. Crosswell , et al., “Drug Recovery and Copay Assistance Program in a Community Cancer Center: Charity and Challenges,” Journal of Oncology Practice/ American Society of Clinical Oncology 15, no. 7 (2019): e628–e635, 10.1200/JOP.19.00016. 31162998

[26] M. V. Ragavan , R. V. Mora , K. Winder , et al., “Impact of a Comprehensive Financial Resource on Financial Toxicity in a National, Multiethnic Sample of Adult, Adolescent/Young Adult, and Pediatric Patients With Cancer,” JCO Oncology Practice 19, no. 2 (2023): e286–e297, 10.1200/OP.22.00350. 36378994

[27] S. M. Kircher , J. Yarber , J. Rutsohn , et al., “Piloting a Financial Counseling Intervention for Patients With Cancer Receiving Chemotherapy,” Journal of Oncology Practice/ American Society of Clinical Oncology 15, no. 3 (2019): e202–e210, 10.1200/JOP.18.00270. 30625023

[28] M. Farrugia , H. Yu , S. J. Ma , et al., “Financial Counseling Is Associated With Reduced Financial Difficulty Scores in Head and Neck Cancer Patients Treated With Radiation Therapy,” Cancers (Basel) 13, no. 11 (2021): 2516, 10.3390/cancers13112516. 34063890 PMC8196601

[29] N. R. Handley , K. Y. Wen , S. Gomaa , et al., “A Pilot Feasibility Study of Digital Health Coaching for Men With Prostate Cancer,” JCO Oncology Practice 18, no. 7 (2022): e1132–e1140, 10.1200/OP.21.00712. 35394806

[30] C. Alacevich , A. M. Abi Nehme , J. H. Lee , et al., “A Point‐Of‐Care Pilot Randomized Intervention to Connect Patients With Cancer‐Induced Financial Toxicity to Telehealth Financial Counseling,” Cancer Causes & Control 35, no. 3 (2024): 393–403, 10.1007/s10552-023-01794-9. 37794203 PMC10872295

[31] V. S. Blinder , A. M. Deal , B. Ginos , et al., “Financial Toxicity Monitoring in a Randomized Controlled Trial of Patient‐Reported Outcomes During Cancer Treatment (Alliance AFT‐39),” Journal of Clinical Oncology 41, no. 29 (2023): 4652–4663, 10.1200/JCO.22.02834. 37625107 PMC10564309

[32] V. Shankaran , T. Leahy , J. Steelquist , et al., “Pilot Feasibility Study of an Oncology Financial Navigation Program,” Journal of Oncology Practice/ American Society of Clinical Oncology 14, no. 2 (2018): e122–e129, 10.1200/JOP.2017.024927. 29272200

[33] C. Lambert , S. Legleitner , and K. LaRaia , “Technology Unlocks Untapped Potential in a Financial Navigation Program,” Oncology Issues 34, no. 1 (2019): 38–45, 10.1080/10463356.2018.1553420.

[34] G. Sadigh , K. Gallagher , J. Obenchain , et al., “Pilot Feasibility Study of an Oncology Financial Navigation Program in Brain Cancer Patients,” Journal of the American College of Radiology 16, no. 10 (2019): 1420–1424, 10.1016/j.jacr.2019.07.014. 31585660 PMC6779332

[35] K. Watabayashi , J. Steelquist , K. A. Overstreet , et al., “A Pilot Study of a Comprehensive Financial Navigation Program in Patients With Cancer and Caregivers,” Journal of the National Comprehensive Cancer Network 18, no. 10 (2020): 1366–1373, 10.6004/jnccn.2020.7581. 33022646

[36] A. Bell‐Brown , T. Hopkins , K. Watabayashi , et al., “A Proactive Financial Navigation Intervention in Patients With Newly Diagnosed Gastric and Gastroesophageal Junction Adenocarcinoma,” Supportive Care in Cancer 32, no. 3 (2024): 189, 10.1007/s00520-024-08399-1. 38400905 PMC10894103

[37] D. A. Parikh , G. M. Rodriguez , M. Ragavan , et al., “Lay Healthcare Worker Financial Toxicity Intervention: A Pilot Financial Toxicity Screening and Referral Program,” Supportive Care in Cancer 32, no. 3 (2024): 161, 10.1007/s00520-024-08357-x. 38366165

[38] M. E. Charles , L. M. Kuroki , A. A. Baumann , et al., “A case study of adapting a health insurance decision intervention from trial into routine cancer care,” BMC Research Notes 15, no. 1 (2022), 10.1186/s13104-022-06189-8. PMC9463661 36088371

[39] M. C. Politi , R. L. Grant , N. P. George , et al., “Improving Cancer Patients' Insurance Choices (I Can PIC): A Randomized Trial of a Personalized Health Insurance Decision Aid,” Oncologist 25, no. 7 (2020): 609–619, 10.1634/theoncologist.2019-0703. 32108976 PMC7356712

[40] A. M. Tarnasky , G. N. Tran , J. Nicolla , et al., “Mobile Application to Identify Cancer Treatment‐Related Financial Assistance: Results of a Randomized Controlled Trial,” JCO Oncology Practice 17, no. 10 (2021): e1440–e1449, 10.1200/OP.20.00757. 33797952 PMC8791821

[41] J. S. Edward , L. E. McLouth , M. K. Rayens , L. P. Eisele , T. S. Davis , and G. Hildebrandt , “Coverage and Cost‐Of‐Care Links: Addressing Financial Toxicity Among Patients With Hematologic Cancer and Their Caregivers,” JCO Oncology Practice 19, no. 5 (2023): e696–e705, 10.1200/OP.22.00665. 36888937 PMC10414719

[42] E. R. Park , A. C. Kirchhoff , K. Donelan , et al., “Health Insurance Navigation Tools Intervention: A Pilot Trial Within the Childhood Cancer Survivor Study,” JCO Oncology Practice 20, no. 7 (2024): 953–963, 10.1200/OP.23.00680. 38471048 PMC11292596

[43] B. Thom , N. Arora , C. Benedict , et al., “Using Real‐World Data to Explore the Impact of One‐Time Financial Grants Among Young Adult Cancer Survivors,” Journal of Adolescent and Young Adult Oncology 12, no. 6 (2023): 912–917, 10.1089/jayao.2022.0188. 37852000 PMC10739788

[44] M. C. Politi , R. W. Yen , G. Elwyn , et al., “Encounter Decision Aids Can Prompt Breast Cancer Surgery Cost Discussions: Analysis of Recorded Consultations,” Medical Decision Making 40, no. 1 (2020): 62–71, 10.1177/0272989X19893308. 31829111

[45] Department of Health and Human Services , “Medical Expenditure Panel Survey Home,” Agency for Healthcare Research and Quality, https://meps.ahrq.gov/mepsweb/.

[46] “Facit Cost,” FACIT Group, https://www.facit.org/measures/facit‐cost.

[47] U.S. Department of Health and Human Services , “Patient‐Reported Outcomes Measurement Information System (PROMIS),” National Institutes of Health, https://commonfund.nih.gov/patient‐reported‐outcomes‐measurement‐information‐system‐promis.

[48] K. M. Esselen , A. Gompers , M. R. Hacker , et al., “Evaluating Meaningful Levels of Financial Toxicity in Gynecologic Cancers,” International Journal of Gynecological Cancer 31, no. 6 (2021): 801–806, 10.1136/ijgc-2021-002475. 33858954 PMC9728957

[49] K. L. Corrigan , S. Fu , Y. S. Chen , et al., “Financial Toxicity Impact on Younger Versus Older Adults With Cancer in the Setting of Care Delivery,” Cancer 128, no. 13 (2022): 2455–2462, 10.1002/cncr.34220. 35417565 PMC9177670

[50] K. R. Yabroff , E. C. Dowling , G. P. Guy , et al., “Financial Hardship Associated With Cancer in the United States: Findings From a Population‐Based Sample of Adult Cancer Survivors,” Journal of Clinical Oncology 34, no. 3 (2015): 259–267, 10.1200/JCO.2015.62.0468. 26644532 PMC4872019

