# Supplementary material for: A Scoping Review of Interventions to Address Financial Toxicity in Pediatric and Adult Patients and Survivors of Cancer
Source: Cancer Med. 2025 Apr 18;14(8):e70879. doi: 10.1002/cam4.70879 (PMC12006751; doi:10.1002/cam4.70879)
Supplement: Supplementary file 1 — Data S1. [file CAM4-14-e70879-s001.docx]

**Supplemental**

**Search Strategy**

Total: 2687 Duplicates: 1174 Unique: 1513

Ovid Medline 881 results on 05/09/24

Ovid MEDLINE(R) ALL <1946 to May 08, 2024>

1 exp financial stress/ or ((financial or economic) adj3 (toxicity or hardship* or burden* or toxicit* or adversity or pressure* or stress* or distress* or strain* or challenge* or deficit or challenge*)).mp. 44702

2 exp neoplasms/ or Oncolog*.mp. or tumor*.mp. or tumour*.mp. or cancer*.mp. or neoplasm*.mp. or malignan*.mp. or adenocarcinoma*.mp. or carcinoma*.mp. or melanoma.mp. or sarcoma*.mp. or leukemia*.mp. or lymphoma*.mp. or melanoma.mp. or glioma*.mp. or neuroblastoma.mp. or rhabdomyosarcoma.mp. or multiple-myeloma.mp. or osteosarcoma.mp. or retinoblastoma.mp. or chordoma.mp. or ependymoma.mp. or estesioneuroblastoma.mp. or mesothelioma.mp. or papillomatosis.mp. or paraganglioma.mp. or pheochromocytoma.mp. or blastoma.mp. or thymoma.mp. 5586447

3 Intervention*.ti. or ((intervention* or screening) adj18 (financ* or cost)).ti,ab. or patient-navigation.mp. or (preauthorization or pre-authorization).mp. or (financ* adj3 (train*

or navigat* or guid* or help* or assist* or resource* or conversation* or education or tailor* or strateg* or cope or coping or program* or manag*)).mp. or (cost adj3 (cope or coping or calculator or conversation* or understand* or program* or manag* or discuss*)).mp. or counseling.mp. or counselling.mp. or ((transportation or co-pay* or copay* or deductible) adj3 (assist* or voucher*)).mp. or exp "Referral and Consultation"/ 534998

4 1 and 2 and 3 1028

5 limit 4 to (english language and yr="2013 -Current") 881

Pubmed 103 results on 5/20/24

(((financial-toxicity[tiab] or financial-hardship*[tiab] OR financial-stress*[tiab] OR financial-distress*[tiab] OR financially-stressed[tiab] OR financial-challenge*[tiab] OR financially-challenge*[tiab] OR financially-distressed[tiab] OR Financial-Resource-Strain[tiab]) AND (Intervention*[title] OR solution*[tiab] OR training[tiab] OR navigation[tiab] OR financial-resource*[tiab] OR conversation[tiab] OR financial-education[tiab] OR program*[title] OR cost-calculator*[tiab] OR cost-coping[tiab] OR financial-counsel*[tiab] OR economic-consult*[tiab] OR financial-consult*[tiab] OR financial-toxicity[title])) AND (Oncolog*[tiab] or tumor*[tiab] or tumour*[tiab] or cancer*[tiab] or neoplasm*[tiab] or malignan*[tiab] or adenocarcinoma*[tiab] or carcinoma*[tiab] or melanoma[tiab] or sarcoma*[tiab] or leukemia*[tiab] or lymphoma*[tiab] or melanoma[tiab] or glioma*[tiab] or neuroblastoma[tiab] or rhabdomyosarcoma[tiab] or multiple-myeloma[tiab] or osteosarcoma[tiab] or retinoblastoma[tiab] or chordoma[tiab] or ependymoma[tiab] or estesioneuroblastoma[tiab] or mesothelioma[tiab] or papillomatosis[tiab] or paraganglioma[tiab] or pheochromocytoma[tiab] or blastoma[tiab] or thymoma[tiab])) NOT (medline[Filter]) AND (english[Filter])

Embase.com 932 results on 05/20/24

1 ‘financial stress’/exp or (financial near/5 (toxicity or hardship* or burden* or toxicit* or adversity or pressure* or stress* or distress* or strain* or challenge* or deficit or challenge*)):ti,ab,kw,de

2 Oncolog*:ti,ab,kw or tumor*:ti,ab,kw or tumour*:ti,ab,kw or cancer*:ti,ab,kw or neoplasm*:ti,ab,kw or malignan*:ti,ab,kw or adenocarcinoma*:ti,ab,kw or carcinoma*:ti,ab,kw or melanoma:ti,ab,kw or sarcoma*:ti,ab,kw or leukemia*:ti,ab,kw or lymphoma*:ti,ab,kw or melanoma:ti,ab,kw or glioma*:ti,ab,kw or neuroblastoma:ti,ab,kw or rhabdomyosarcoma:ti,ab,kw or multiple-myeloma:ti,ab,kw or osteosarcoma:ti,ab,kw or retinoblastoma:ti,ab,kw or chordoma:ti,ab,kw or ependymoma:ti,ab,kw or estesioneuroblastoma:ti,ab,kw or mesothelioma:ti,ab,kw or papillomatosis:ti,ab,kw or paraganglioma:ti,ab,kw or pheochromocytoma:ti,ab,kw or blastoma:ti,ab,kw or thymoma:ti,ab,kw

3 Intervention*:ti or ((intervention* or solution*) near/18 (financ* or cost)):ti,ab,kw or (screen* near/4 financial):ti,ab,kw,de OR patient-navigation:ti,ab,kw,de or (preauthorization or pre-authorization):ti,ab,kw,de or (financ* near/3 (train* or navigat* or guid* or help* or assist* or resource* or conversation* or education or tailor* or strateg* or cope or coping or program* or manag*)):ti,ab,kw,de or (cost near/3 (cope or coping or calculator or conversation* or understand* or program* or manag* or discuss*)):ti,ab,kw,de or counseling:ti,ab,kw,de or counselling:ti,ab,kw,de or ((transportation or co-pay* or copay* or deductible) near/3 (assist* or voucher*)):ti,ab,kw,de

4 #1 and #2 and #3

5 'financial toxicity':ti AND trial*:ti,kw,de

6 #5 AND# 2

7 #4 OR #6

8 #7 AND ([article]/lim OR [article in press]/lim OR [data papers]/lim OR [editorial]/lim OR [erratum]/lim OR [letter]/lim OR [note]/lim OR [review]/lim OR [short survey]/lim OR [preprint]/lim) AND [english]/lim AND [2013-2024]/py

Web of Science Core Collection 753 results on 5/20/24

1 (TS= ((financial OR monetary) near/5 (toxicity or hardship* or burden* or toxicit* or adversity or pressure* or stress* or distress* or strain* or challenge* or deficit or challenge*)))

2 TI= (Oncolog* or tumor* or tumour* or cancer* or neoplasm* or malignan* or adenocarcinoma* or carcinoma* or melanoma or sarcoma* or leukemia* or lymphoma* or melanoma or glioma* or neuroblastoma or rhabdomyosarcoma or multiple-myeloma or osteosarcoma or retinoblastoma or chordoma or ependymoma or estesioneuroblastoma or mesothelioma or papillomatosis or paraganglioma or pheochromocytoma or blastoma or thymoma) OR AB= (Oncolog* or tumor* or tumour* or cancer* or neoplasm* or malignan* or adenocarcinoma* or carcinoma* or melanoma or sarcoma* or leukemia* or lymphoma* or melanoma or glioma* or neuroblastoma or rhabdomyosarcoma or multiple-myeloma or osteosarcoma or retinoblastoma or chordoma or ependymoma or estesioneuroblastoma or mesothelioma or papillomatosis or paraganglioma or pheochromocytoma or blastoma or thymoma)

3 (TI= (Intervention* OR solution*)) or TS=((intervention* or solution* OR counsel*) near/7 (financ* or cost)) or TS=(screen* near/4 financial) OR TS=(patient-navigation OR counseling or counselling) or TS= (preauthorization or pre-authorization) or TS= (financ* near/3 (train* or navigat* or guid* or help* or assist* or resource* or conversation* or education or tailor* or strateg* or cope or coping or program* or manag*)) or TS=(cost near/3 (cope or coping or calculator or conversation* or understand* or program* or manag* or

discuss*)) or TS=((transportation or co-pay* or copay* or deductible) near/3 (assist* or voucher*))

4 #1 and #2 and #3

5 #4 refine by Publication Years: 2013 through 2024; Languages: English’ Document Types: Aricle or Revew Article or Early Access or Editorial Material or Letter or Book Chapters or Correction

Clinicaltrials.gov 18 results on 5/20/24

( AREA[ConditionSearch] cancer OR AREA[TitleSearch] cancer ) AND AREA[InterventionSearch] financial stress
